# School-based promotion of physical literacy: a scoping review

**DOI:** 10.3389/fpubh.2024.1322075

**Published:** 2024-03-08

**Authors:** Martin Grauduszus, Lennart Koch, Stefanie Wessely, Christine Joisten

**Affiliations:** Department for Physical Activity in Public Health, Institute of Movement and Neurosciences, German Sport University Cologne, Cologne, Germany

**Keywords:** physical literacy, school-based interventions, children, physical activity promotion, health promotion

## Abstract

**Introduction:**

The role of physical activity in children’s healthy development is undisputed, with school-based interventions being seen as a priority. The promotion of physical literacy (PL) seems to be promising due to its holistic approach, combining physical, cognitive, and affective domains. To develop recommendations for possible measures, we compiled existing literature on existing school-based PL interventions.

**Methods:**

Five databases (MEDLINE, Web of Science, SPORTDiscus, ERIC, and PsycInfo) were searched between July 6 and July 10, 2023, by combining the terms “physical literacy,” “school,” “program,” “workshop,” “intervention,” and “curriculum” as well as a manual search. Records were screened in a two-stage process by two independent authors using *a priori* criteria. Eligible studies concerned PL interventions in the school context. The included records were sorted according to school type/population, structure, content, PL domains addressed, and evaluation.

**Results:**

In total, 706 articles were found through the database search and an additional 28 articles through the manual search. After removing duplicates, 502 publications remained, which were screened by title and abstract, leaving 82 full texts. These were cut down to 37 articles describing 31 different programs (19 in primary schools, eight in secondary schools, one in both primary and secondary schools, and three unspecified). Most interventions were conducted during physical education classes (*n* = 12). All three PL domains were addressed by five interventions, while 11 interventions solely concerned the physical domain. In addition, 21 interventions evaluated their effects on PL. Most evaluations showed small to moderate but inconsistent effects on several PL-related constructs (e.g., self-efficacy, motivation, movement skills). Interventions incorporating all three domains reported positive effects on physical competence and enjoyment.

**Discussion:**

Although there is a growing body of data related to school-based PL promotion, their effects and practical application remains relatively underdeveloped: study designs, study quality, PL assessments, and results are heterogeneous. Corresponding research adhering to the holistic approach of PL will be crucial in clarifying the potential lifelong role of PL in promoting physical activity, increasing health and well-being and to actually enable development of recommendations for action.

## 1 Introduction

Physical activity and exercise play a central role in the healthy physical, psychosocial, cognitive, and emotional development of children and adolescents (1–3). However, school-aged children tend to engage in sedentary behavior and excessive use of audiovisual media. Steene-Johannessen et al. (4) integrated 30 studies conducted between 1997 and 2014 into a systematic review that used accelerometry to measure physical activity levels and sedentary behavior in children aged 2–9.9 years and adolescents aged 10–18 years. Notably, only 29% of the children and adolescents were classified as being sufficiently physically active. Boys were more active in all age categories. The beginning of the age-related decrease in or leveling off of physical activity and the increase in sedentary behavior seemed to occur roughly at the age of 6–7 years. The COVID-19 pandemic significantly worsened this trend, leading to a reduction in children’s physical activity of between 11 and 91 min a day (5).

Due to the numerous negative consequences associated with physical inactivity, such as motor deficits, obesity, and weight gain, effective counter measures are warranted. In this context, schools emerge as an ideal setting: the fact that young people spend a significant proportion of their time in schools and actively participate in school activities makes them a strategic and accessible setting for targeted interventions (6). However, although a range of measures has been introduced in schools to promote physical activity and reduce sedentary behavior (7–9), there is still no gold standard for effective interventions. Following a systematic Cochrane review including 89 studies, representing data from 66,752 study participants, the increase in the time spent engaging in moderate to vigorous physical activity through school-based physical activity intervention is small to non-existent (mean difference = 0.73 min/day; 95% confidence interval = 0.16–1.30 min/day). The authors emphasize that considering the diversity of effects, the potential for bias, and the generally modest magnitude of effect, the results should be interpreted cautiously (10).

Factors influencing participation in physical activity are multicomponent encompassing social environment and intrapersonal level, among others (11). Therefore, there is a need to implement more comprehensive strategies targeting daily life and living environments as well as additional factors such as the intrinsic motivation and self-efficacy of children and adolescents to initiate and maintain an active or healthy lifestyle. A promising approach in this context is the holistic concept of physical literacy (PL) developed by Whitehead (12, 13). Within this concept a cognitive domain (knowledge and understanding of the physical and psychological effects of sports and exercise), an affective domain (integrating various constructs like motivation and exercise-related self-efficacy and self-confidence), and a physical domain (movement, sports participation, motor skills, and basic movement skills) was summarized. According to her, these domains are interrelated and form the basis of a lifelong active lifestyle (14). A cross-sectional Danish study explored the associations between adolescents’ PL and their emotional and social well-being and whether these associations are mediated by sports and exercise participation. Positive associations were observed between PL, well-being, and exercise participation (15). Additionally, Carl et al. (16) described positive effects of PL interventions on individual domains as well as on physical activity behavior. However, this review mainly analyzed the effects on the PL or their individual domains and did not relate them to the respective setting or the intervention content. Given that appropriate measures in schools can significantly contribute to lifelong physical activity, a more in-depth analysis of such interventions within the school setting is essential to develop appropriate recommendations. Therefore, we conducted a scoping review to answer the following questions: What theoretical PL concepts are school-based PL interventions based on? How are PL interventions implemented in everyday school life, in terms of program length, frequency, and duration of individual units? Which assessment instruments were used to measure the effects of the interventions on PL? What effects do school-based PL interventions have on PL outcomes?

## 2 Methods

This scoping review was conducted according to the methodological framework elaborated by Arksey and O'Malley (17). This article is based on the Preferred Reporting Items for Systematic Reviews and Meta-Analyses Extension for Scoping Reviews (PRIMSA-ScR) (18).

The search strategy was based on Whitehead’s (12) definition and the three domains of PL. The cognitive domain incorporated knowledge and understanding of the changes in the body and psyche due to movement. The affective domain covered the areas of motivation, self-efficacy, and self-confidence. The physical domain encompassed motor skills, movement behavior, and basic movement skills.

All school types were addressed: primary, secondary, and high school. The distinction between primary and secondary school was defined by the school system of the country of origin of the intervention. Secondary schools were defined as any school with an International Standard Classification of Education (ISCED) level-3 qualification (19) at the maximum, which includes, for example, American High Schools.

### 2.1 Search strategy and selection process

The following five databases were searched for articles published by July 6, 2023: MEDLINE (via PubMed), Web of Science, SPORTDiscus, ERIC, and PsycInfo. The search was conducted by combining the terms “physical literacy,” “school,” “program,” “workshop,” “intervention,” and “curriculum.” Details on the specific search strategies used on each database can be found in the appendix (Supplementary Table S1). In addition, the reference lists of systematic literature reviews were searched to identify relevant publications. If study protocols were included, a search was conducted for the published results of the study. Where possible, inaccessible full texts were requested from the corresponding author by email three times.

Publications were included if the following *a priori* criteria were met: (i) a PL intervention/program/workshop/curriculum implementation (hereafter referred to as an intervention) or an intervention designated as such was used; (ii) the intervention targeted school children, or the effects of the intervention on school children were examined; (iii) the intervention took place in a school context; and (iv) the publication was written in English or German. Publications were excluded if (i) the PL intervention was aimed at kindergarten children, preschool children, university students, school staff, or parents; (ii) the PL intervention did not take place in a school context; (iii) the publication was a conference paper or scientific poster or was not written in English or German.

Studies were selected using the online program Rayyan (20). Duplicates were first removed automatically and then manually. Two authors (M.G. and L.K.) independently and blindly screened the identified publications against the inclusion and exclusion criteria in two steps: (i) title and abstract screening and (ii) full-text screening. Disagreements were discussed at the end of each step. If no consensus could be reached, a third author (C.J.) decided.

**Figure 1:**
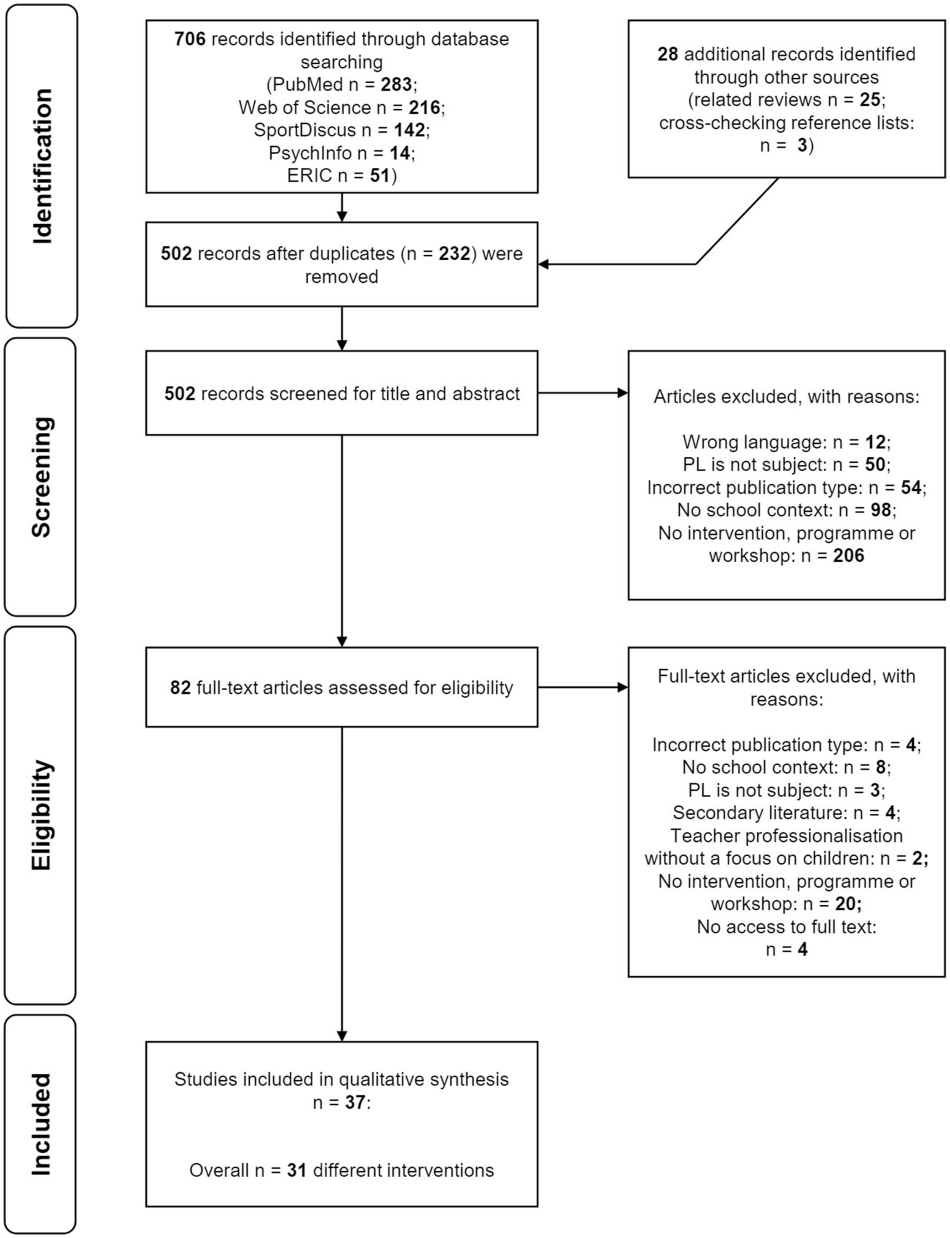
PRISMA flow diagram presenting the process of study selection.

The search and selection processes were documented in a PRIMSA flow chart (Figure 1) (21).

### 2.2 Data extraction

A standardized extraction table was developed *a priori* and was initially tested for applicability and completeness using five publications. This pilot test demonstrated that the extraction table could capture basic publication data, intervention classification and description data, evaluation results, and evaluation classification data. A complete list of the extracted data items can be found in the appendix (Supplementary Table S2).

### 2.3 Synthesis

The implementation of PL into the school routine was recorded based on the time of everyday school life when the intervention was conducted (after school, physical education (PE), multi-component, other) and the type of school (primary and secondary school). The structure was assessed by length (in weeks), frequency (in sessions/week), and duration (in minutes/unit).

The realization of the three PL domains in each intervention was assessed using the following criteria. The criteria for the affective and cognitive domains were considered to be fulfilled as soon as they were mentioned or described in the intervention description; example for fulfilled affective domain: “[…] by engaging the students in an experience that would provide individual challenges, also known as positive challenges, they would concurrently develop aspects of the affective domain of physical literacy. Not only would students experience these optimal challenges, but in doing so they could develop feelings of positive affect such as fun and enjoyment, which would foster motivation,” (22); example for fulfilled cognitive domain: “The cognitive aspect of the psychological domain was specifically worked on in the circuits through understanding movements and using feedback and knowledge of results to improve,” (22). The physical domain criterion was fulfilled if at least one additional physical activity session took place (e.g., active breaks) or a new concept was implemented in regular PE lessons (e.g., the SAMPLE-PE intervention by Rudd et al. (23): Children explored objects in the PE hall. Activities with changing constraints were played. No demonstration and feedback were provided. Instead, children reflected using questioning strategies or observed their peers. Questioning fostered an external focus of attention). Conversely, carrying out regular PE lessons did not fulfill the physical domain criterion.

Additionally, to be able to consider the effectiveness of the interventions on PL outcomes, study designs, assessment instruments, and reported results were obtained, if available.

## 3 Results

### 3.1 Literature search and study characteristics

The search of the online databases returned 706 articles, with another 28 articles identified through manual searching (see Figure 1). After duplicates were removed, the titles and abstracts of the remaining 502 sources were screened. In the next step, the full text of 82 articles was assessed for eligibility. In total, 37 articles describing 31 different interventions met the inclusion criteria.

Eight interventions were conducted in Canada, seven in the United States, three each in Germany and Wales, two in Hong Kong, and one each in Australia, England, Ireland, Scotland, Slovakia, Spain, and Turkey. For one intervention, the country of origin could not be determined.

### 3.2 Underlying theoretical physical literacy concepts within the interventions

All of the interventions identified in this study (*n* = 31) referred to a PL model. Most frequently, Whitehead (12, 13, 24) was cited when deriving a definition (*n* = 14). The definition of the International Physical Literacy Association (IPLA), which is closely connected to the perspective of Whitehead, was referred to seven times: “Physical literacy can be described as the motivation, confidence, physical competence, knowledge and understanding to value and take responsibility for engagement in physical activities for life” (25). Canada’s Consensus Statement, which aligns with the definition established by the IPLA, was described once (26). A review by Edwards et al. (27) was mentioned twice; it presents a summary of existing PL definitions, with the main result that approximately half of the approaches are based on a monist/holistic PL perspective. The definition proposed by the Aspen Institute was also mentioned twice: “Physical Literacy is the Ability, Confidence, and desire to be Physically Active for Life” (28). One intervention presented its own definition: “Physical literacy is a part of the ontogenetic development of the individual […]. A physically literate person should have adequate motor abilities, skills, and knowledge, including a positive attitude to physical activities, and is able to take responsibility for his own health” (29). In four instances, no specific details were provided regarding the definition applied in the intervention.

In relation to the theoretical construct PL five interventions focused all three domains. Two domains were addressed by 15 measures each (physical and affective: *n* = 9; physical and cognitive: *n* = 5; affective and cognitive: *n* = 1). The physical domain alone was addressed by eleven interventions.

### 3.3 Physical literacy assessments

Overall, 21 interventions were evaluated in terms of isolated PL domains (Tables 1–4). The effects on PL as an overarching construct were assessed five times, using the Canadian Assessment of Physical Literacy (*n* = 1), the second version of this assessment (*n* = 3), and the Passport for Life tool (*n* = 1). PL self-perception was evaluated using the Physical Literacy Assessment for Youth Self (PLAYself) questionnaire (*n* = 3).

**Table 1:** Identified interventions conducted during physical education lessons.

Author, year: Project	Country	Participants			Intervention characteristics			Structure of intervention	Content of intervention				Study design	Construct: instrument	Results
		N	Age (mean ± SD) [years]	Female [%]	Length [wk]	Frequency [PLS/wk]	Duration [min/PLS]		Description	Cognitive	Affective	Physical			
*Primary school*															
Borzikova et al. (2020)	Slovakia	84	6.8 ± 0.4	–	24	1.25	60–72	One session included 6 physical activities or movement games. Intervention sessions were additional to standard PE.	Physical exercises and activities with non-traditional equipment and psychomotor games.	No	No	Yes	RCT	Basic motor competencies: MOBAK (“Motorische Basiskompetenz”).	Basic motor competencies: post-intervention IG favored, *p* < 0.01 (unpaired *t*-test, IG 11.95 ± 2.09, CG 7.20 ± 2.72).
Coyne et al. (2018): Athletics Canada’s Grassroots RJTW Program	Canada	310	10.5 ± 1.0 Range: 7–12	50.3	10	2	40	Running, jumping, and throwing programs of 3 weeks each.	Track-and field-inspired games, activities, and skill challenges.	No	No	Yes	Non-controlled study	PL: Canadian Assessment of Physical Literacy	PL: pre-post-intervention time effect IG, *p* < 0.001, Cohen’s *d* = 0.303 (paired *t*-test, pre intervention 61.7 ± 10.4, post intervention 65.0 ± 11.4).
Deutsch et al. (2022): Best Warm-up Activities	USA	75	9.0 ± 1.0	60.0	4	1.5	30	15 min one “physical-best” or traditional warm-up +15 min activity games.	“Physical-best” warm-up: (i) Jumping Frenzy: stations with instruction cards for various jump rope activities and stretches. At each rest station, children self-assess what activities were most intense and beneficial to physical health. (ii) Artery Avengers: fill an opponent’s hula hoop (arteries) with yarn balls (fat from food) while keeping their hula hoop empty. (iii) Clean the Beach: collecting beanbags (trash) and placing them in hula hoops (trash can) using various locomotor movements (walking on all fours, tiptoes, hopping on one foot). After the activity is over, students identify which body parts’ muscular strength was developed by each locomotor movement.	Yes	No	Yes	Quasi-experimental controlled intervention trial.	Health-related knowledge: multiple choice questionnaire.	Health-related knowledge: pre-post-intervention time effect, *p* = 0.02, small effect (repeated measure ANOVA).
Johnstone et al. (2017): Go2Play active play	Scotland	189	7.0 ± 1.1	56.1	20	2	60	One session: 30 min of structured games and 30 min of free play.	The first half of the session was fun, inclusive, and active games focused on improving a specific FMS area. Each session concentrated on one FMS area so that a broad range of skills was covered over the intervention period. The second half was free play, which allowed children to practice what they learned in the first half of the session and/or to create and play their own games using a variety of traditional equipment, such as balls, beanbags, cones, hoops, etc.	No	Yes	Yes	Quasi-experimental controlled intervention trial.	Motor skills: Test of Gross Motor Development (TGMD-2).	Motor skills: time*group effect IG favored, *p* < 0.04, pre-post-intervention time effect IG, *p* < 0.01 (repeated measure ANOVA).
Kriellaares et al. (2019): Circus Arts Instruction in Physical Education (CAI-PE)	Canada	211	10.1 ± 0.8	55	20 (a), 52 (b), 10 (c) (d.o.s)	2 (a), 3 (b), 1 (c) (d.o.s)	60 (a, b), 50 (c) (d.o.s)	–	Wide range of circus disciplines from the five major circus families (clowning, manipulation, equilibriums, aerials, and acrobatics). Artistic movement expression, technical variations in expression, and choice of progressions were fostered to encourage self-challenges and ownership of movement.	No	Yes	Yes	Quasi-experimental controlled intervention trial.	No evaluation of PL outcomes.	
Rudd et al. (2020) and Crotti et al. (2021): SAMPLE-PE	England	360	5.9 ± 0.3	55	15	2	60	Three 5-week phases: dance, gymnastics, ball sports.	At the beginning of each lesson, coaches invited children to explore the PE hall and the different objects within the environment. The lesson continued with activities representative of game, sport, or performance situations where coaches introduced variability by changing constraints. Coaches did not provide demonstrations or feedback during activities. Alternatively, they invited children to reflect using questioning strategies or to observe their peers. Coaches also used questioning to foster an external focus of attention in the child to infuse variability in the task and channel children’s learning.	No	Yes	Yes	Protocol	No data available	
Stoddart et al. (2021): PLitPE	Canada	131	9.7 ± 0.6	49.6	9	3	25	Two sets of circuit stations on two separate days (Circuit 1: 8 skills; Circuit 2: 6 skills), with a third day specifically focused on locomotor patterns (e.g., skip, gallop, crossovers).	Each station had a laminated poster that provided an image, performance cues for the movements, and instructions for the task. Students were provided with choices that enabled them to modify activities based on their own skill level and desired challenge. The remainder of the PE class was spent teaching content working towards other curricular outcomes. Depending on what the teachers had previously covered in the curriculum, during the second half of class, teachers taught content such as dance, flag football, track and field, and other topics. The circuits were adapted when possible to allow for transfer.	Yes	Yes	Yes	Quasi-experimental controlled intervention trial	PL self-perception: PLAYself. Physical competence: PLAYfun	PL self-perception: no significant pre-post-intervention time effects, post-intervention IG favored in one subscale, *p* < 0.039 (unpaired *t*-test, IG 423.9 ± 89.5, CG 390.1 ± 87.6) Physical competence: pre-post-intervention time effect IG, *p* < 0.001, Cohen’s *d* = 0.88 (paired *t*-test, pre intervention 42.3, post intervention 49.4), post-intervention IG favored, *p* < 0.001, Cohen’s *d* = 1.04 (unpaired *t*-test, IG 49.4 ± 7.1, CG 40.0 ± 2.9).
Wainwright et al. (2018): Foundation Phase	Wales	49	Range: 5–6	55.1	44	–	–	–	The Foundation Phase is a play-based, holistic, child-centered approach to education for children aged 3 to 7, underpinned by childhood well-being. Curriculum documentation advocates the use of indoor and outdoor spaces that are exciting, fun, stimulating, and safe and promote discovery and independence. The “use of the outdoors for learning” is one of four key features of the Foundation Phase, along with “play and active learning,” “child-initiated learning,” and “focused adult-led sessions.”	No	Yes	Yes	Quasi-experimental controlled intervention trial	Motor skills: Test of Gross Motor Development, second edition (TGMD-2). Confidence: Pictorial Scale of Perceived Competence and Social Acceptance (PSPCSA). Motivation: Leuven Involvement Scale for Young Children (LIS-YC).	Motor skills: time effect, *p* < 0.001, *η*^2^ = 0.66 (repeated measures ANOVA) Confidence: pre-post-intervention time effect, *p* = 0.016 (paired *t*-test) Motivation: no description of calculation for intervention effects.
Wainwright et al. (2019): Foundation Phase	Wales	164	5.5 ± 0.6	–	8	2	45							Motor skills: Test of Gross Motor Development, version 3 (TGMD-3)	Motor skills: Pre-post-intervention percentage change IG favored, *p* < 0.001 (unpaired *t*-test, IG 35% ± 19%, CG 2% ± 25%)
*Secondary school*															
Alagul et al. (2012)	Turkey	25	-	-	4	-	80	40 min of salsa and 40 min of PL.	Physical moves of salsa dance were practiced, such as fundamental steps, moving figures, and paired choreography. PL involved abilities like reading, writing, speaking, and listening practices.	No	No	Yes	Qualitative study	No PL evaluation	
Liu and Chen (2021)	USA	226	12.2 ± 0.7	53.3	8	0.5	20–30	One session: (i) motivational module, (ii) informational module.	High-and low-performing PL students were separated during workshops. The activities to implement the motivational module included instruction, communication, and encouragement, where pedagogical skills were used to facilitate student engagement. To complete the series of activities during the motivational module, the student participants (a) shared with others their fun experiences, challenges/barriers, and social experiences related to physical activities, (b) received encouragement to participate in physical activities where they can seek fun, (c) worked together to provide possible solutions for others to overcome difficulties in performing these activities, and (d) received strategies for better socializing with others. The informational module was subsequently delivered with instruction and demonstration concerning the knowledge of health-related fitness and PA, tips to improve movement skills, health-related fitness, and behavioral strategies.	Yes	Yes	No	Non-controlled study	PL: Canadian Assessment of Physical Literacy-2 (CAPL-2)	PL subscales: time*group effect (groups of high- vs. low-performing PL levels) for the subscale Behavioral domain, *p* < 0.01, *η*^2^ = 0.36 (ANCOVA)
Strobl et al. (2020)	Germany	233	14.66 ± 1.27	54.94	52	–	–	(i) Teachers participated in a participatory planning process to conceptualize evidence-based PE lessons; (ii) they then implement these lessons in physical education.	The learning outcomes should follow a holistic understanding of health and fitness: psychosocial aspects, short-and long-term benefits of physical activity for the improvement of physical fitness at school as well as in their spare time, activity-related behavior in terms of risk factors, injuries and illnesses, and knowledge and understanding of how social and mental well-being are interrelated with physical activity.	Yes	No	Yes	Quasi-experimental controlled intervention trial.	Health-related knowledge and understanding: multiple choice questionnaire.	Health-related knowledge and understanding: post-intervention IG favored, *p* < 0.001, *η*^2^ = 0.066 (ANCOVA adjusted for type of school, sex, baseline).
Haible et al. (2019) and Rosenstiel et al. (2022): Promotion of physical activity-related health competence in PE (GEKOS)	Germany	841	14.20 ± 0.51	51.13	6	1	90	-	The special feature of the GEKOS intervention is the combination of its methodical approach to addressing knowledge, skills, abilities, and motivation and its content focus on health and fitness. The lessons emphasized health and fitness, both theoretically and practically, using the two main topics of perception of physical load and control of physical load and physical training. The individual lessons focused on content that included (1) the perception of physiological responses to PA, (2) the perception and measurement of heart rate, (3) the perception and measurement of perceived exertion, (4/5) health-related fitness (strength training and cardiovascular endurance), and (6) the application of skills and knowledge.	Yes	No	Yes	RCT	PL evaluation planned	

d.o.s, depending on school; min, minutes; IG, intervention group, PA, physical activity; PE, physical education; PL, physical literacy; PLS, physical literacy session; SD, standard deviation; wk, week.

**Table 2:** Other identified interventions.

Author, year: project	Country	Implementation	Participants			Intervention characteristics			Structure of intervention	Content of intervention				Study design	Construct: Instrument	Results
			N	Age (mean ± SD) [years]	Female [%]	Length [wk]	Frequency [PLS/wk]	Duration [min/PLS]		Description	Cognitive	Affective	Physical			
*Primary school*																
Eveland-Sayers et al. (2022)	United States	During school time	92	–	–	6	1	30	One session: warm-up (6 min), jumping (15 min), throwing (8 min), homework (1 min).	Movement skills focused on locomotor skills (running mechanics, various skips, shuffling, carioca/grapevine, running pace, starts, hurdles, broad jump, hops, bounding, proper landing, and balance) and ballistic skills (throwing). Homework included practicing, physical challenges (e.g., cross-legged sit-to-stand without hands on the ground), bodyweight exercises (e.g., pushups, squats), and stretching.	No	No	Yes	Non-controlled study	Self-efficacy for physical activity: Children’s Self-Perception of Adequacy in and Predilection for Physical Activity (CSAPPA).	Self-efficacy for physical activity: time*group interactions favored children with higher BMI, *p* = 0.03, *η*^2^ = 0.097 (ANCOVA).
Wright et al. (2020): Job Embedded Professional Development (JEPD)	Canada	Professional development/PE	551 Pupils 15 Teachers	IG 7.9 ± 1.7; Range: 4.7–10.8 CG 7.6 ± 1.6; Range: 4.8–11.0 Teachers: Range: 25–44.	Pupils: 45.8; Teachers: 87.0.	10	1	30	–	Games and activities that developed competence in movement skills and built confidence, motivation, and knowledge of physical activity in the children. The activities and skills covered included teaching cues for running, jumping, throwing, and catching, as well as other movements such as galloping, hopping, striking, and dribbling.	Yes	Yes	Yes	Quasi-experimental controlled intervention trial	Physical competence: PLAYbasic	Physical competence: time*group effect IG favored for one item, *p* < 0.05; pre-post-intervention time effect IG for all five items, *p* < 0.05 (repeated measure ANOVA).
Mendoza-Muñoz et al. (2022): Active breaks (AB)	Spain	Breaks	57	10.3 ± 0.4 Range: 8–12	50.9	4	7	15	One session: warm-up (2–3 min), games and activities (15 min), cool-down (2–3 min).	Warm-up: meeting-time and mobility exercises. Cooperative and competitive games (catch the flag, rock paper scissors, dodge ball, fox hospital, card games) with meeting-time and mobility exercises of increasing difficulty. Cool-down: relaxation exercises, time for sharing experiences.	No	Yes	Yes	Quasi-experimental controlled intervention trial.	PL: Canadian Assessment of Physical Literacy-2.	PL: pre-post-intervention time effect IG, *p* < 0.001, (ANOVA, pre intervention 61.19 ± 11.96, post intervention 68.30 ± 10.85); post-intervention IG favored, *p* < 0.017 (ANOVA, IG 68.30 ± 10.85, CG 60.72 ± 11.90).
^*^Sum et al. (2018): Physical Education Continuing Professional Development (PE-CPD)	Hong Kong	Professional development	–	–	–	32	1.6	60	PE lessons taught by teachers who participated in the physical education continuing professional development intervention.	Teaching and learning domain (24 h): pedagogical workshop of fundamental movement, Teaching Games for Understanding (TGfU), and sports education; PE homework; using IT in PE. Student development domain (8 h): seminar on understanding students’ diverse needs; workshops and sharing session on planning and organization of student development sports activities. School development domain (6 h): exemplary sharing of home–school collaboration on parent-related school activities; roles of PE and sports as promoting factors of the school culture and school image. Professional relationships and services domain (12 h): workplace learning through action research; institutional learning to facilitate understanding of research findings and best practices.	Yes	No	Yes	Protocol	PL evaluation planned	
*Secondary school*																
Pullen et al. (2020)	Wales	Summer school	46	Range: 11–14	56.5	6	1.3	–	–	Strength-and conditioning-based activities for athletic motor skill competencies: to stimulate strength adaptations, resistance was provided using body weight, resistance bands, or medicine balls. Basic resistance training equipment was incorporated into games, challenges, or short periods of teaching to learn techniques. Games and challenges utilized an individualized, constraint-led approach by manipulating task and environmental constraints. Many exercises were integrated into games to make the intervention enjoyable and engaging for the pupils.	No	Yes	Yes	Quasi-experimental controlled intervention trial	Motivation to exercise: behavioral regulation in exercise. Physical self-efficacy: Perceived Physical Ability Scale for Children	Motivation to exercise: pre-post-intervention time effect male IG in one subscale, *p* < 0.05 (paired and unpaired *t*-test, Mann–Whitney *U*-test); Physical self-efficacy: No significant results for IG (paired and unpaired *t*-test, Mann–Whitney *U* test).
^*^Sum et al. (2018): Physical education continuing professional development (PE-CPD)	Hong Kong	Professional development	–	–	–	32	1.6	60	PE lessons taught by teachers who participated in the physical education continuing professional development intervention.	Teaching and learning domain (24 h): pedagogical workshop on fundamental movement, Teaching Games for Understanding (TGfU), and sports education; PE homework; and using IT in PE. Student development domain (8 h): seminar on understanding students’ diverse needs; workshops and sharing session on planning and organization of student development sports activities. School development domain (6 h): exemplary sharing of home–school collaboration on parent-related school activities; roles of PE and sports as promoting factors of the school culture and school image. Professional relationships and services domain (12 h): workplace learning through action research; institutional learning to facilitate understanding of research findings and best practices.	Yes	No	Yes	Protocol	PL evaluation planned	

^*^Sum et al. (30): This intervention was carried out in primary and secondary schools. Therefore, it is mentioned in both sections. IG, intervention group; Min, minutes; PL, physical literacy; PLS, physical literacy session; SD, standard deviation; wk, week.

**Table 3:** Identified interventions conducted as after-school interventions.

Author, year: project	Country	Participants			Intervention characteristics			Structure of intervention	Content of intervention				Study design	Construct: Instrument	Results
		N	Age (mean ± SD) [years]	Female [%]	Length [wk]	Frequency [PLS/wk]	Duration [min/PLS]		Description	Cognitive	Affective	Physical			
*Primary school*															
Caldwell et al. (2022a) and Caldwell et al. (2022b): Build Our Kids’ Success (BOKS) programming	Canada	14	9.3	55.0	8	–	70	Contains different elements: full-length physical activity plans (20–45 min), short movement breaks (1–10 min), and movement-based games, activities, and resources for school or at-home use.	The full-length physical activity plans include a warm-up activity (i.e., adventure run, BOKS Says), running-related activity (e.g., running relays, musical run), skill of the week (i.e., planks, sprints), game (i.e., crab walk, soccer, red light-green light), cool down (i.e., deep breaths, full-body stretch), and a BOKS Bits nutrition talk. The short movement breaks are designed to keep children active throughout the activity and may include activities such as an ABCWorkout, Bingo Burst, or BOKS Says.	No	No	Yes	Non-controlled study	Physical activity enjoyment: Physical Activity Enjoyment Scale (PACES). PL self-perception: PLAYself.	Only post-intervention descriptive results.
Carl et al. (2023): PLACE	Germany	–	Range: 8–11	-	24 in each of three cycles (2 pilot studies, 1 main study).	1	60–90	Sessions will be driven by the concept of PL (physical, affective, social domain), with direct links between theory, content, and actual movement.	Rule-based games primarily via ball games and racket sports. The aesthetic input will focus on dancing and acrobatics, and fitness will be dominantly targeted via endurance-oriented games or in the context of parkour. Theory-based inputs in each session (i.e., content knowledge, rules, strategies and planning, tactics, awareness, as well as purposing and reasoning). Transferring and supporting principles of motivation, autonomy, enjoyment, self-awareness, and confidence. Application of diverse group compositions and game arrangements.	Yes	Yes	Yes	Protocol	PL evaluation planned.	
Mandigo et al. (2018): Teaching Games for Understanding (TGfU) for the PlaySport Intramural Program	Canada	22	–	72.7	8	3–4	60	One session consisted of a game activity to introduce the main objectives of the lesson, a movement development, and a culmination, which provided the participants with an opportunity to integrate what they had learned throughout the lesson into a game activity.	Overall, there were seven target/individual game sessions, three net/wall game sessions, five striking/fielding game sessions, and 10 territorial game sessions delivered during this time period.	Yes	No	Yes	Non-controlled study	PL: Passport for Life	PL subscales: pre-post-intervention time effect for subscales Balance *p* < 0.001, Cardiovascular *p* = 0.001, Diverse environments *p* = 0.003, and Diverse interests *p* < 0.002 (paired *t*-test, balance: pre intervention 2.23 ± 0.69, post intervention 2.91 ± 0.43; Cardiovascular: pre intervention 1.57 ± 0.60, post intervention 2.43 ± 1.08; Diverse environments: pre intervention 2.68 ± 0.37, post intervention 2.97 ± 0.41; Diverse interests: pre intervention 2.74 ± 0.75, post intervention 3.00 ± 0.75); for eight subscales, no significant results.
*Secondary school*															
Grimes et al. (2022) and Lightner et al. (2023): Move More, Get More	USA	116	IG: 13.4 ± 1 CG: 13.8 ± 1.0	39.7	36	1–3 (based on school)	60–120	One session consisted of warm-up (10 min), activity (40–100 min), and cool-down (10 min) activities and sports rotated every 2 weeks.	Variety of sports and skills necessary to participate in diverse sports; snowball recruitment and focus on team-oriented sports; scrimmages and step challenges using accelerometers. Incentives were used. Skill development and inclusiveness and limited over-competitiveness by implementing no-cut policies. Activity types included traditional sports (basketball, soccer, football, etc.), team-based activities (capture the flag, dodgeball, etc.), dance, yoga, and others.	No	Yes	Yes	Post-intervention only design	Physical competence: PLAYbasic	Physical competence: post-intervention IG favored, *p* = 0.004 (unpaired *t*-test IG 75.62 ± 13.14, CG 50.71 ± 19.73).
*No information*															
Bremer et al. (2020)	Canada	90	IG: 9.1 ± 1.4 CG: 10.5 ± 1.8 Range: 7–13	46.67	12	5	30	Each skill block lasted 3 days. One session consisted of 15 min of learning fundamental movement skills and 15 min of an active game.	Skill block: focused on learning and practicing a different set of fundamental movement skills (e.g., jumping, throwing, catching). Active game: incorporating the day’s movements. All active games were chosen from the PlaySport activities. The level of difficulty of both the skill stations and the active game progressed over the course of the 3-day skill block and more generally over the 12-week intervention.	Yes	Yes	Yes	RCT	Physical competence: PLAYfun. PL self-perception: PLAYself. Self-efficacy, motivation, enjoyment, perceived knowledge: questionnaire.	Multiple linear regression models adjusted for age, sex, baseline score: Physical competence: experimental group *p* = 0.10, *r*-squared = 0.728. Self-efficacy: experimental group *p* = 0.85, *r*-squared = 0.541. Motivation: experimental group *p* = 0.14, *r*-squared = 0.330. Enjoyment: experimental group *p* = 0.03, *r*-squared = 0.391. PL self-perception: experimental group *p* = 0.90, *r*-squared = 0.289.
Crozier et al. (2022): PL-focused afterschool activity programs (ASAPs)	Canada	29	IG: 8.3 ± 1.3 CG: 8.6 ± 1.7 Range: 5–12	55.2	24	5	180	–	PL-focused afterschool activity program that promotes healthy active lifestyles to children via introducing and facilitating a wide range of sports and athletic opportunities.	No	No	Yes	Quasi-experimental controlled intervention trial	Aerobic capacity: PACER, Motor skills: Test of Gross Motor Development–2 (TGMD-2)	Aerobic capacity: No significant pre-post-intervention time and post-intervention group effects (paired and unpaired *t*-test, Wilcoxon test). Motor skills: pre-post-intervention time effect for subscale object control, *p* = 0.024. No significant post-intervention group effects
Lewis et al. (2013): Growing Young Moves	No information	–	–	–	–	2	–	–	Various physical education activities in the gymnasium space.	No	No	Yes	Project description	No PL evaluation	

IG, intervention group; Min, minutes; PL, physical literacy; PLS, physical literacy session; RCT, randomized controlled trial; SD, standard deviation; wk, week.

**Table 4:** Identified interventions classified as using a multi-component approach.

Author, year: Project	Country	Participants			Intervention characteristics			Structure of intervention	Content of intervention				Study design	Construct: instrument	Results
		N	Age (mean ± SD) [years]	Female [%]	Length [wk]	Frequency [PLS/wk]	Duration [min/PLS]		Description	Cognitive	Affective	Physical			
*Primary school*															
Hulteen et al. (2023): Peer Leadership for Physical Literacy (PLPL)	Canada	227	–	–	10	2	30	Two phases: (i) development of leadership among Grade 6/7 peer leaders; (ii) Grade 6/7 peer leaders deliver a 10-week movement skills program to the younger Grade 3/4 students.	Each movement skill session focused on one of six object-control skills (i.e., catching, overhand throwing, underhand throwing, kicking, dribbling, and a two-handed strike with a baseball bat). Each of these skills was taught between three (catch, overarm throw, two-handed strike, dribble) and four times (underarm throw, kick) throughout the 10-week program.	No	No	Yes	RCT	Motivation: Self-determined motivation questionnaire. Perceived competence: questionnaire. Self-concept: Physical Self-Description Questionnaire-Short Version. Motor skills: Test of Gross Motor Development, third edition.	Multiple linear regression models adjusted for sex, baseline score: Motivation: experimental group *p* = 0.236, *r*-squared = 0.228. Perceived competence: experimental group *p* = 0.181, *r*-squared = 0.361. Self-concept: experimental group *p* = 0.153, *r*-squared = 0.347. Motor skills: maximal throw speed: experimental group *p* = 0.128, *r*-squared = 0.770; Throw-catch combination: experimental group *p* = 0.870, *r*-squared = 0.263; throw process score: experimental group *p* = 0.839, *r*-squared = 0.497.
Li et al. (2021) and Li et al. (2022): Stand+Move	Hong Kong	79	SSPLAY: 9.7 ± 0.7 PLAY: 9.6 ± 0.6 CG: 9.6 ± 0.6	SSPLAY: 62.5 PLAY: 55.6 CG: 60.7	13	10 (active breaks)	15 (active breaks) Continuous sit-stand desks	Children participated in a play activity during recess time followed by several minutes of cool-down.	PLAY: unstructured outdoor interactive games led by PE interns (skipping rope, shuttlecock, kicking, hide-and-seek). SSPLAY: additional height-adjustable sit-stand desks in the classroom. The goal was to use the stand desk for at least 1 h/day.	No	No	Yes	RCT	PL: Canadian Assessment of Physical Literacy-2 Chinese.	PL subscales: time*group effects favored IG post intervention for subscales Physical competence *p* = 0.02 and Daily behavior *p* = 0.004. No significant results at 3-month follow-up.
Telford et al. (2020): Physical Education and Physical Literacy (PEPL)	Australia	303	IG: 10.41 ± 0.39 CG: 11.14 ± 0.39	51	33	Continuous	Continuous	An additional PE lesson each week together with four activity sessions of 15–40 min in the schoolyard.	Classroom teacher professional development; in-class PE assistance; provide PE lesson and activity plans as required; provide lesson plans for physical activity breaks; support, encourage, and motivate classroom teachers to deliver PE lessons; conduct physical activity sessions during school lunch breaks focusing on fundamental movement skills; provide teachers with strategies and activities to increase physical activity during breaks and before and after school; encourage students to join an extracurricular sports club.	No	Yes	Yes	RCT	Motor skills: Test of Gross Motor Development, second edition (TGMD-2). Physical self-perception: Children and Youth – Physical Self-Perception Profile (CY-PSPP). Physical activity enjoyment: Shortened-Physical Activity Enjoyment Scale (S-PACES).	Multiple linear regression models adjusted for study condition, sex: Motor skills: object control: IG value of *p* = 0.008; locomotor: IG value of *p* = 0.471. Physical self-perceptions: sport competence: IG value of *p* = 0.013; physical condition: IG value of *p* = 0.466; physical self-worth: IG value of *p* = 0.551. Physical activity enjoyment: IG value of *p* = 0.737.
Gavigan et al. (2023): Moving Well-Being Well (MWBW)	Ireland	925	7.55 Range: 6–10	–	8	(i) 2 PE classes; (ii) five active classroom activities; (iii) one home activity sheet.	(i) 30; (ii) 5–10	Three main components: (i) FMS-based PE classes, (ii) active classroom activities, (iii) home activity sheet.	The content of the three main components focused on just three locomotor (hop, skip, and jump) and three object-control skills (kick, catch, and throw).	No	No	Yes	Qualitative study	No PL evaluation	
Driscoll and Linker (2022)	United States	–	–	–	–	–	–	The homework (home fun) should reinforce the skills learned in PE in other subjects or at home with family and friends.	The homework (home fun) should include enjoyable physical activity. The purpose is to reinforce concepts, knowledge, and skills (locomotor skills: hopping, galloping, running, sliding, skipping, leaping, yoga/stretching) learned in PE outside regular PE class (in other subjects, at home with family and friends).	No	Yes	Yes	Project description	No PL evaluation	
*Secondary school*															
Shawley (2016): Creating Healthy Active Minds for Personal Success (CHAMPS)	United States	–	–	–	–	–	–	Two PE semester blocks, each consisting of 4–7 weeks separated into four blocks. One block provides two 49-min lessons, followed by two 72-min lessons.	(i) Students wear a pedometer daily and download steps at the end of each class. Students use this data for goal setting. (ii) Each block offers health and fitness content (health-related fitness knowledge, intensity levels, measuring MVPA, fitness testing, program design, technology and apps, skill-related fitness, circuit training) and motor skills and activities (football or rugby, ultimate frisbee, tennis, choice week, soccer, pickleball, disc golf, weight room and functional fitness, social dance, basketball, weight room fitness plans, volleyball, health lab). Health and fitness content is provided in the first half of the long lessons.	Yes	Yes	Yes	Project description	No PL evaluation	
Altieri (2019): Get Ready Program	United States	6	–	–	52	–	–	–	The Get Ready program engages students in physical activity in the school’s weight room, gym, and dance studio. The program’s elements are designed to help the students with their physical development through physical activities and help them take personal and social responsibility in this physical activity setting. Gradually, the students are empowered to be able to run the program with less and less direction from the Get Ready facilitators. Eventually, the goal is for them to become more and more confident to be able to coach themselves and even other students through these sessions.	No	No	Yes	Qualitative study	No PL evaluation	

IG: intervention group; Min, minutes; PL, physical literacy; PLS, physical literacy session; SD, standard deviation; wk, week.

In 13 interventions, the effects on the physical domain were assessed via motor test batteries: motor skills (TGMD-2: *n* = 4; TGMD-3: *n* = 2), physical competence (PLAYbasic: *n* = 2; PLAYfun: *n* = 3), aerobic capacity (PACER: *n* = 1), and basic motor competencies (MOBAK: *n* = 1). In eight interventions, the effects on the affective domain were assessed with constructs such as motivation (Leuven Involvement Scale for Young Children: *n* = 1; Behavioural Regulation in Exercise: *n* = 1; subscale from adapted behavioral regulation and psychological need satisfaction scales: *n* = 1; self-developed: *n* = 1), confidence (Pictorial Scale of Perceived Competence and Social Acceptance: *n* = 1), self-efficacy (Children’s Self-Perception of Adequacy in and Predilection for Physical Activity: *n* = 1; Perceived Physical Ability Scale for Children: *n* = 1), self-concept (Physical Self-Description Questionnaire-Short Version: *n* = 1), perceived competence (subscale from adapted behavioral regulation and psychological need satisfaction scales: *n* = 1), and self-perception (PLAYself: *n* = 4). In two interventions, the effects on the cognitive domain were assessed using multiple-choice questionnaires about health-related knowledge. No information about validation was obtained for one questionnaire, and the other was validated in-house.

### 3.4 Implementation in physical education lessons

Of the identified interventions, 12 were implemented during PE lessons, they are presented in Table 1 (22, 23, 29, 31–42).

#### 3.4.1 Structure, domains, and effects in physical education: primary school

Eight interventions were conducted during PE lessons at primary schools (22, 23, 29, 32–34, 36, 37, 41, 42) with a number of participants ranging from 49 to 360, a mean age between 5.5 and 10.5 years, and proportion of female participants of 49.6–60%. The length of the intervention varied between 4 and 52 weeks. The frequency of PL sessions ranged between 1 and 3 sessions per week. The duration of one PL session ranged from 30 to 72 min.

Only one intervention addressed all three domains (22), while five interventions targeted two. Specifically, four covered the affective and physical domains (23, 36, 37, 41, 42), and one the cognitive and physical domains (34). The other three interventions focused solely on the physical domain during PE (29, 32).

Six interventions were evaluated. The PLitPE intervention demonstrated large positive effects on physical competencies compared to the control group (*p* < 0.001, Cohen’s *d* = 1.04). It focused on all three domains through the practice of movement skills using a playful approach. Additionally, knowledge about movement terminology was obtained to address the cognitive domain (22). Interventions targeting one or two PL domains showed various small to moderate positive effects on health-related knowledge, motor skills, and confidence levels compared to the pre-intervention assessments (Table 1) (29, 34, 36, 41, 42).

#### 3.4.2 Structure, domains, and effects in physical education: secondary school

Four interventions were conducted at secondary schools (31, 38, 40), with between 25 and 841 participants, a mean age range from 12.2 to around 14.7 years, and a proportion of female of 51.1–54.9%. The lengths of the interventions were 4, 6, 8, and 52 weeks. The frequency of PL sessions was 0.5 and 1 session per week (missing information for two interventions). The duration of one PL session varied between 20 and 90 min.

No intervention included all domains. Three interventions addressed two domains: two covered the cognitive and physical domains (35, 39, 40), one the affective and cognitive domains (38), and the remaining intervention only focused on the physical domain (31).

Two interventions were evaluated. A medium-sized positive effect (*p* < 0.001, *η*^2^ = 0.066) on health-related knowledge and understanding compared to the control group was found for an intervention addressing the cognitive and physical domain through lessons implemented specially to address health-related knowledge and understanding. One example of this was that pupils carried out research on swimming-specific strength training in preparation for swimming classes. They presented and carried out their findings in class (40). The effects of another intervention were evaluated in a non-controlled study and are shown in Table 1.

### 3.5 Implemented as other school-based approaches

Other school-based approaches included qualifications of teachers and the implementation of content during lessons [*n* = 2; (30, 43)], break-time activities [*n* = 1; (44)], and a summer-school program [*n* = 1; (45)]. The setting for one approach was not further described but took place during school time (Table 2) (46). The intervention by Sum et al. (30) was carried out in primary and secondary schools and is therefore mentioned in both of the next two sections.

#### 3.5.1 Structure, domains, and effects in other school-based approaches: primary school

Of these five interventions, four were conducted at primary schools (30, 43, 44, 46). The number of participants ranged from 57 to 551. Among the two interventions with available data, the mean age was 7.8 and 10.3 years. The percentages of female participants were 45.8 and 50.9%. The length of the primary school interventions ranged from 4 to 32 weeks. The PL sessions took place between one and seven times a week, with each session lasting between 15 and 60 min.

One intervention addressed all three PL domains (43). Of the remaining three interventions, two targeted two domains: one the affective and physical domains (44) and the other the cognitive and physical domains (30). One intervention focused solely on the physical domain (46).

Three interventions were evaluated. Positive significant effects on an overall PL score compared to the control group were reported for the “active breaks” intervention (*p* = 0.017). It addressed the affective and physical domains by getting children to engage in game-based physical activity during their breaks (44). Notably, the “Job embedded professional development” intervention addressing all three domains reported a positive effect on only one of five physical competence items compared to the control group (motor skill overhand throw, *p* < 0.05) (43). Further effects are presented in Table 2.

#### 3.5.2 Structure, domains, and effects in other school-based approaches: secondary school

Two interventions were implemented at a secondary school (30, 45). While data is missing for one intervention, the other had 57 participants, with an age range from 11 to 14 years and 56.5% of female participants. The lengths of the interventions were 6 and 32 weeks, with an average of 1.3 and 1.6 sessions per week. In the intervention with 1.6 sessions per week, each session lasted 60 min.

Both interventions addressed two PL domains: one the affective and physical domains (45) and the other the cognitive and physical domains (30). Only one intervention was evaluated, displaying significantly positive effects on one subscale of the motivation to exercise compared to the pre-intervention assessment. There were no effects on physical self-efficacy compared to the pre-intervention assessment and the control group (45).

### 3.6 Implemented as after-school programs

Seven interventions were conducted as after-school programs (Table 3) (47–56).

#### 3.6.1 Structure, domains, and effects in after-school programs: primary school

Three after-school interventions took place at primary schools (48–51, 56). Two interventions provided information about participants. There were 14 participants in one (female = 55%, mean age = 9.3 years) and 22 in the other (female = 72.7%, no information about age). The lengths of the interventions ranged from 8 to 24 weeks, with a frequency between 1 and 3.5 sessions per week and a single session duration of 60–75 min.

One intervention addressed all three PL domains (50). One intervention focused on two PL domains, namely, the cognitive and physical domains (56). The last intervention targeted solely the physical PL domain (48, 49).

Two interventions were evaluated. The intervention by Mandigo et al. reported the most relevant positive effects on four out of 12 PL subscales, namely, balance (*p* < 0.001), cardiovascular (*p* = 0.001), diverse environments (*p* = 0.003), and diverse interests (*p* = 0.002), compared to the pre-intervention assessment. It addressed the physical and cognitive PL domains through an intervention drawing on the Teaching Games for Understanding approach (56). Further results are shown in Table 3.

#### 3.6.2 Structure, domains, and effects in after-school programs: secondary school

One intervention was developed for secondary school children (53, 55). The intervention involved 116 participants, with a mean age of 13.6 years, and 39.7% female participants. The length of the intervention was 36 weeks, with two 90-min sessions per week.

The “move more, get more” intervention incorporated the affective and physical domain through step challenges using accelerometers, among others. A positive effect on physical competence was reported compared to the control group (*p* = 0.004) (53, 55).

#### 3.6.3 Structure, domains, and effects of additional after-school programs

For three interventions, no information about the type of school was provided (47, 52, 54). Two of them addressed children and youth between 5 and 12 years old and between 7 and 13 years old, respectively, with 29 and 90 participants. The shares of female participants were 46.7 and 55.2%. The lengths of the interventions were 12 and 24 weeks, respectively, with five sessions per week each. The length for one session was 30 and 180 min, respectively. For the third intervention, very limited information was available (54).

One intervention addressed all three domains (47), whereas the other two focused solely on the physical domain (52, 54).

Two interventions were evaluated (47, 52). A positive effect on enjoyment was achieved by the intervention studied by Bremer et al. (*p* = 0.03, r-squared = 0.391). It addressed all three PL domains through daily 30-min skill blocks. During the first 15 min of each block, fundamental movement skills were taught, and the remaining time was dedicated to active play. Interestingly, no effects on physical competence (*p* = 0.1), self-efficacy (*p* = 0.85), motivation (*p* = 0.14), and PL self-perception (*p* = 0.9) compared to the control group were found (47). The effects of the other evaluated after-school program are presented in Table 3.

### 3.7 Implemented as multi-component interventions

Seven interventions were classified as using a multi-component approach that required more than one setting at a time (Table 4) (57–64).

#### 3.7.1 Structure, domains, and effects in multi-component interventions: primary school

Five interventions took place in primary schools (58–62, 64). Although four of these were described in detail, one only gathered information about content and not about the formal structure and participants. Among these four interventions, the number of participants ranged from 79 to 925, with a mean age of 9.7 to 10.8 years. The information about the proportion of female participants in the intervention was only given for two studies, standing at 51.0 and 59.1% (62, 64). The lengths of the interventions ranged from 8 to 33 weeks. One intervention was implemented with continuous measures. The others were implemented through 2, 2, and 10 sessions per week with a duration of 15, 15, and 30 min per session, respectively.

Two interventions addressed two PL domains, namely, the affective and physical domains (58, 64). The other three focused solely on the physical PL domain (59–62).

Three interventions were evaluated (60, 62, 64). The most pronounced positive effects on the motor skill of object control (*p* = 0.008) and the physical self-perception of sport competence (*p* = 0.013) were achieved by the “physical education and physical literacy” intervention (64). This intervention addressed the affective and physical PL domains and consisted of an additional PE lesson that emphasized the development of fundamental movement skills. Physical activity sessions were also conducted during lunch breaks and after school. Noteworthy is the positive effect (*p* = 0.004) on physical competence on the daily behavior subscale of a PL assessment in the “Stand+Move” intervention, which vanished at the 3-month follow-up (61, 62). Further results are shown in Table 4.

#### 3.7.2 Structure, domains, and effects in multi-component interventions: secondary school

For the two interventions in secondary schools, no information on participants, length, frequency, or duration was obtained (57, 63).

Regarding the content structure, one intervention incorporated all three PL domains, with students wearing pedometers and using their step data to set goals. In addition, the PE curriculum was divided into blocks offering health and fitness content (e.g., health-related fitness knowledge), motor skills, and activities (63). The other intervention only focused on the physical domain (57). Neither intervention evaluated PL outcomes.

## 4 Discussion

To the best of the authors’ knowledge, this is the first scoping review to compile interventions that promote PL in school settings. A total of 31 interventions were identified across 37 papers, most of which took place in primary schools during PE lessons. The interventions were highly heterogeneous in terms of sample size, content, duration, and frequency. All three domains were covered by only five interventions, whereas nearly all studies addressed motor skills, focusing on a diverse range of physical activities. About half of them were designed to promote the joy of movement and, thus, motivate students to increase physical activity. The cognitive domain was rarely addressed.

About two-thirds of interventions were evaluated regarding PL outcomes (21 out of 31). Here, too, there was great heterogeneity in terms of study quality, measurement methods, and intervention content, making comparisons difficult. Small/medium effects, if any, were found for interventions, mostly addressing the physical and affective domains. When an intervention concerned all three PL domains, the effects were promising regarding physical competence and enjoyment. One intervention showed large effects on physical competencies. However, other PL outcomes (e.g., self-efficacy, PL self-perception, motivation) were not affected. Additionally, no long-term effects were measured. Therefore, it remains unclear how sustainable the effects of interventions are and how they correspond to the idea of a lifelong learning process.

It is hardly surprising that PL is mainly taught in primary school and especially in PE lessons. Early encouragement is intended to lay the foundations for a lifelong physical activity. On top of this, PE, besides its purely physical component, plays a critical role in promoting an active and healthy lifestyle by imparting knowledge and understanding to students and motivating them (65).

However, the extent to which this is sustainable remains to be determined. In addition to the lack of data, tracking evidence-based progress in this context is methodologically challenging as decades of study are often necessary to assess the sustainable (health) effects of interventions in childhood. Because this is hardly feasible, surrogate parameters are frequently used to evaluate an intervention’s effectiveness, such as motor skills performance, measures of fitness, academic performance, and health parameters (body composition, lipids, blood pressure, mental health, etc.). But even here only small effects become clear. Based on a meta-analysis of 20 studies integrating data about 6,621 children and adolescents aged 4–18 years, Hartwig et al. (66) reported a very small “increase” in cardiorespiratory fitness of 0.47 mL/kg/min and in moderate-intensity physical activity of approximately 1 min a day for school based interventions that are not based on holistic approaches like PL.

Therefore, even though strategies like the Global Action Plan on Physical Activity of the World Health Organization have already been developed, the effects are (very) small. A rethink in terms of skills/literacy promotion as fundamental for physical activity behavior seems to make sense. The idea of improving skills/literacy has also been discussed in health. Chrissini and Panagiotakos (67) called for the inclusion of health literacy in health policy agendas as an essential and decisive strategy to empower individuals to take action. To enhance health literacy, people should be empowered to comprehend and apply information related to healthy lifestyles, particularly in the context of self-care. In turn, a healthy lifestyle supports health literacy by improving the cognitive and physical resources needed to process health information. This way of thinking is applicable to the promotion of PL. Corresponding initiatives could be useful tools in the (early) fight against non-communicable diseases, especially in the school setting. For example, teaching skills through play, including physical activity as part of self-care, may help students to increase their relevant knowledge, gain (positive) experience, and adapt their behavior accordingly (mod. after) (68). In other words, a holistic and competence-oriented approach to promoting physical activity is necessary. In Germany, this can already be found in a broader sense in school curricula with the dual mandate of “education in and through sport” (69). In the literature, however, an underlying theoretical framework is often missing (70).

Nevertheless, what conclusions can be drawn from the contents? The aim of the review was to develop appropriate recommendations for the promotion of PL in schools. Due to the heterogeneity of the studies described above and the largely non-holistic implementation of the interventions, we are unable to develop concrete recommendations at this state of research. More high-quality studies that implement the holistic PL concept are needed as a basis for recommendations. Nevertheless, it can be tentatively hypothesized that primary school environments and PE classes present promising venues for the promotion of PL. Diverse and playful forms of movement such as dance, fitness, games, gymnastics, individual activities, and outdoor activities seem to contribute to the development of different competencies. However, emphasis should not solely be placed on advancing the physical domain, but also on nurturing affective and cognitive domains to align with a holistic perspective, as delineated by Whitehead. The incorporation into teacher training programs holds promise for yielding the most profound effects, fostering an accompanying mindset and favorable disposition toward PL education. As concerns lifelong learning, the role of educators is to teach individuals to make healthy, active choices throughout their lives and to understand that physical activity is not limited to one school subject or the school setting.

### 4.1 Strengths and weaknesses

Our scoping review has several strengths and weaknesses. The methodological approach of a scoping review allows for a methodologically clear and high-quality presentation of the existing literature. We attempted to present the data, including the interventions and their effects, in as much detail as possible to derive recommended actions. However as mentioned above, the described interventions were highly heterogeneous. Moreover, in several cases, not all measures were evaluated, or an evaluation was not (yet) available. Possible influencing factors, such as students’ neighborhoods or their families’ levels of education, were also missing, which made an evaluation or derivation of good practice models difficult. Another challenge was categorizing the interventions in terms of which PL domains were addressed and which were not. In doing so, we followed the Whiteheadian definition and relied on what the authors reported in their publications. This was challenging because some authors briefly mentioned individual domains, while others provided detailed and comprehensive information. This point should be taken into consideration when assessing the interventions presented in this study.

## 5 Conclusion

The promotion of PL in schools appears to be a promising approach as a basis for a lifelong active (and healthy) lifestyle and as a means to combat non-communicable diseases. Currently, PL promotion mostly occurs in PE classes in primary schools through a variety of playful activities. The implementation in school curricula and the qualification of teachers are encouraging, but the effects of these efforts have not yet been tested. This is largely because although more data on PL promotion is becoming available, the application of this concept to the context of physical activity and health promotion is not well established in the scientific literature. Further research is therefore needed on the nature and direction of the relationship between PL, its individual domains, physical activity, and health to clarify the possible lifelong role of PL in promoting physical activity, increasing health and well-being, and to actually enable development of recommendations for action.

## Data availability statement

The original contributions presented in the study are included in the article/Supplementary materials, further inquiries can be directed to the corresponding authors.

## Author contributions

MG: Conceptualization, Data curation, Formal analysis, Investigation, Methodology, Visualization, Writing – original draft, Writing – review & editing. LK: Conceptualization, Data curation, Investigation, Methodology, Visualization, Writing – original draft, Writing – review & editing. SW: Conceptualization, Supervision, Writing – review & editing. CJ: Conceptualization, Project administration, Supervision, Writing – original draft, Writing – review & editing.
