# Supplementary material for: School-based promotion of physical literacy: a scoping review
Source: Front Public Health. 2024 Mar 8;12:1322075. doi: 10.3389/fpubh.2024.1322075 (PMC10959127; doi:10.3389/fpubh.2024.1322075)
Supplement: Supplementary file 1 [file Table_1.docx]

Supplementary Material

**Supplementary Table 1.** Search strategies used for the respective databases.

| **Database** | **Date of search** | **Search strategy** | **Numbers of records found** |
| --- | --- | --- | --- |
| MEDLINE (via PubMed) | 06th July 2023 | 1: ("physical literacy") | 347 |
|  |  | 2: (school) | 7128347 |
|  |  | 3: ("primary school") | 11933 |
|  |  | 4: ("middle school") | 7080 |
|  |  | 5: ("high school") | 41838 |
|  |  | 6: (#2) OR (#3) OR (#4) OR (#5) | 7128347 |
|  |  | 7: (#1) AND (#6) | 292 |
|  |  | 8: (program) | 1856727 |
|  |  | 9: (workshop) | 2008791 |
|  |  | 10: (intervention) | 1856727 |
|  |  | 11: (curriculum) | 10304526 |
|  |  | 12: (#8) OR (#9) OR (#10) OR (#11) | 12265296 |
|  |  | **13: (#7) AND (#12)** | **283** |
| Web of Science | 06th July 2023 | 1: ALL=("physical literacy") | 614 |
|  |  | 2: ALL=(school) | 11613540 |
|  |  | 3: ALL=("primary school") | 24776 |
|  |  | 4: ALL=("middle school") | 17003 |
|  |  | 5: ALL=("high school") | 77297 |
|  |  | 6: #2 OR #3 OR #4 OR #5 | 11613540 |
|  |  | 7: #1 AND #6 | 370 |
|  |  | 8: ALL=(program) | 6057286 |
|  |  | 9: ALL=(workshop) | 327089 |
|  |  | 10: ALL=(intervention) | 1434884 |
|  |  | 11: ALL=(curriculum) | 142910 |
|  |  | 12: #8 OR #9 OR #10 OR #11 | 7520758 |
|  |  | **13: #7 AND #12** | **216** |
| SPORTDiscus | 06th July 2023 | S1: "physical literacy" | 523 |
|  |  | S2: school | 269262 |
|  |  | S3: "primary school" | 2851 |
|  |  | S4: "middle school" | 3522 |
|  |  | S5: "high school“ | 29707 |
|  |  | S6: S2 OR S3 OR S4 OR S5 | 269262 |
|  |  | S7: S1 AND S6 | 255 |
|  |  | S8: program | 20235 |
|  |  | S9: workshop | 6273 |
|  |  | S10: intervention | 78992 |
|  |  | S11: curriculum | 12984 |
|  |  | S12: S8 OR S9 OR S10 OR S11 | 270965 |
|  |  | **S13: S7 AND S12** | **142** |
| ERIC | 06th July 2023 | 1: ALL=("physical literacy") | 174 |
|  |  | 2: ALL=(school) | 763880 |
|  |  | 3: ALL=("primary school") | 20613 |
|  |  | 4: ALL=("middle school") | 47352 |
|  |  | 5: ALL=("high school") | 154234 |
|  |  | 6: #2 OR #3 OR #4 OR #5 | 763880 |
|  |  | 7: #1 AND #6 | 81 |
|  |  | 8: ALL=(program) | 587615 |
|  |  | 9: ALL=(workshop) | 34812 |
|  |  | 10: ALL=(intervention) | 102631 |
|  |  | 11: ALL=(curriculum) | 234633 |
|  |  | 12: #8 OR #9 OR #10 OR #11 | 78182 |
|  |  | **13: #7 AND #12** | **51** |
| PsycInfo | 10th July 2023 | 1: "physical literacy".ab,hw,id,ot,tc,ti,tm. | 111 |
|  |  | 2: „school“.ab,hw,id,ot,tc,ti,tm. | 468258 |
|  |  | 3: "primary school".ab,hw,id,ot,tc,ti,tm. | 13758 |
|  |  | 4: "middle school".ab,hw,id,ot,tc,ti,tm. | 23139 |
|  |  | 5: "high school“ .ab,hw,id,ot,tc,ti,tm. | 10061 |
|  |  | 6: 2 OR 3 OR 4 OR 5 | 468258 |
|  |  | 7: 1 AND 6 | 34 |
|  |  | 8: „program“.ab,hw,id,ot,tc,ti,tm. | 275420 |
|  |  | 9: „workshop“.ab,hw,id,ot,tc,ti,tm. | 12506 |
|  |  | 10: „intervention“.ab,hw,id,ot,tc,ti,tm. | 338380 |
|  |  | 11: „curriculum“.ab,hw,id,ot,tc,ti,tm. | 77037 |
|  |  | 12: 8 OR 9 OR 10 OR 11 | 614862 |
|  |  | **13: 7 AND 12** | **14** |
