# Supplementary material for: School-based promotion of physical literacy: a scoping review
Source: Front Public Health. 2024 Mar 8;12:1322075. doi: 10.3389/fpubh.2024.1322075 (PMC10959127; doi:10.3389/fpubh.2024.1322075)
Supplement: Supplementary file 2 [file Table_2.docx]

Supplementary Material

**Supplementary Table 2.** Extraction table

| Basic publication data | Publication title |
| --- | --- |
|  | Name of the project |
|  | Name of the author |
|  | E-Mail-Address of the author |
|  | Publication year |
|  | Country |
| Intervention description data | School type |
|  | Implementation type |
|  | Number of participants |
|  | Age of participants |
|  | Sex of participants |
|  | Time period of intervention |
|  | Frequency of intervention sessions |
|  | Duration of intervention session |
| Intervention classification data | Structural design of the intervention session |
|  | Content structure of the intervention session |
|  | Cognitive domain of physical literacy considered |
|  | Affective domain of physical literacy considered |
|  | Physical domain of physical literacy considered |
| Evaluation classification data | Study design |
|  | Evaluation available |
|  | Physical literacy evaluation available |
|  | Name of the physical literacy instrument used |
|  | Physical activity evaluation available |
|  | Name of the physical activity instrument used |
| Evaluation results data | Results regarding physical literacy |
|  | Results regarding physical activity |
|  | Other outcome parameters evaluated |
